# SPREAD AND SUSTAINMENT OF QUALITY IMPROVEMENTS IN VA RURAL PRIMARY CARE CLINICS

**DOI:** 10.1093/geroni/igz038.2776

**Published:** 2019-11-08

**Authors:** Josea Kramer, Claire O’Hanlon, Joe Douglas, Michael N Mitchell, Michael McClean, Shawn Clarke, Carol Callaway-Lane

**Affiliations:** 1 VA Greater Los Angeles Healthcare System GRECC, Los Angeles, California, United States; 2 Center of Innovation, Los Angeles, California, United States; 3 Geriatric Research, Education and Clinical Center, Sepulveda, California, United States; 4 Center of Innovation, Geriatric Research, Education and Clinical Center, Sepulveda, California, United States; 6 Geriatric Research, Education and Clinical Center, Nashville, Tennessee, United States

## Abstract

The VA has invested in developing the skills of its primary care workforce through the longitudinal Geriatric Scholars Program. Now in its 11th year, the program has increased career satisfaction and job retention, standardized provider behaviors, improved clinical decision-making and reduced dispensing of potentially inappropriate medications. The program consists of: intensive coursework in geriatrics; workshop in quality improvement (QI); and initiation of a micro QI projects in the Scholar’s clinic. Electives enable learners to tailor the program to self-identified gaps in knowledge, skills and competencies. This presentation focuses on the sustainment and spread of these QI projects based on a recent survey of Scholars. Differences between rural and urban QI projects are compared. Commonality among rural QI projects is explored based on topic, team composition, and the types of efficiencies gained in clinical and/or organizational processes to improve care for older Veterans living in rural areas.

